# New Updates in Adipocytes and Adipose Tissue: 2nd Edition

**DOI:** 10.3390/life16040619

**Published:** 2026-04-07

**Authors:** Endre Kristóf, Éva Csősz

**Affiliations:** 1 Laboratory of Cell Biochemistry, Department of Biochemistry and Molecular Biology, Faculty of Medicine, University of Debrecen, H-4032 Debrecen, Hungary; 2 Proteomics Core Facility, Department of Biochemistry and Molecular Biology, Faculty of Medicine, University of Debrecen, H-4032 Debrecen, Hungary

## 1. Introduction

Long-term imbalance between energy intake and expenditure, coupled with genetic susceptibility, results in excess adiposity associated with deleterious outcomes across a broad range of cell and tissue types. The progression from a lean to an obese phenotype not only remodels the local adipose tissue microenvironment but also exerts systemic effects on distant organs [1]. Adipocytes can respond to chronic calorie surplus either by hyperplasia or hypertrophy, with the latter being strongly associated with the development of insulin resistance and dysregulation of lipid metabolism [2]. Moreover, a phenotypic shift in macrophage (MΦ) polarization from the anti-inflammatory M2 state, predominant in lean adipose tissues, to the pro-inflammatory M1 state promotes chronic low-grade inflammation, driven by increased secretion of pro-inflammatory cytokines and a concomitant decay in anti-inflammatory adipokines such as adiponectin [3]. The onset of inflammation and alterations in adipokine production are tightly regulated through coordinated interactions between the adipocytes and MΦs, as well as other immune cell types [4].

The skeletal muscle is a complex organ with profound effects on the homeostasis of the entire body. Myocytes produce metabolites and signaling lipids in addition to secreting protein factors (myokines), which are regulated by physical exercise (PE). PE activates AMP-activated protein kinase and peroxisome proliferator-activated receptor γ coactivator-1α in myocytes, therefore improving glucose uptake and mitochondrial biogenesis in skeletal muscle and reducing the abovementioned systemic low-grade inflammation [5]. Some myokines, including irisin, interleukin (IL)-6, and meteorin-like, induce adipocyte browning, thereby enhancing energy expenditure and promoting excess nutrient catabolism [6,7].

This Special Issue contains four original research articles and a comprehensive review, focusing on inflammation, PE, and novel prevention and therapeutic strategies for obesity.

## 2. An Overview of Published Articles

Adipocytes, MΦs, and other stromal–vascular cells within adipose tissue coordinately regulate inflammatory events and adipokine secretion [8]. Savulescu-Fiedler et al. (Contribution 1) comprehensively review molecular events during adipose tissue remodeling in response to excess fat deposition, which drive local and systemic low-grade inflammation, whose triggering cues involve fatty acid release by dying hypertrophic adipocytes, hypoxia, and oxidative stress. In turn, robust pro-inflammatory signaling cascades are activated—primarily the nuclear factor-κB and NLR family pyrin domain containing 3-inflammasome pathways—resulting in immune cell recruitment, M2 to M1 MΦ polarization, and the release of pro-inflammatory cytokines, such as tumor necrosis factor-α, IL-6, IL-12, and IL-23. Moreover, excess adipose tissue expansion and inflammation result in significant changes in the levels of adipokines, which exert paracrine or endocrine effects. Weight loss is associated with a significant decrease in both adipose tissue and systemic inflammation, along with improved cardiometabolic risk. However, residual inflammation may persist, possibly preventing the success of therapeutic interventions aiming at weight reduction and the restoration of metabolic homeostasis.

Several plant species are rich in flavonoids reported to ameliorate obesity-associated metabolic complications, such as inflammation, adipose tissue disfunction, glucose intolerance, ectopic fat deposition, and cardiovascular comorbidities, partially through the modulation of adipokine secretion [9]. Nobushi et al. (Contribution 2) investigated the effects of 15 flavanone derivatives classified into a subfamily of flavonoids during the differentiation of the most commonly used murine preadipocyte cell line, 3T3-L1 cells. 4′-phenylflavanone treatment decreased triacylglycerol accumulation and downregulated the mRNA expression of adipogenic markers in a concentration-dependent manner. The suppression of in vitro adipogenesis may be at least partially attributed to the inhibition of mitotic clonal expansion induced by 4′-phenylflavanone administration. Further studies are required to validate these findings in primary adipocyte and in vivo models, as well as to explore the complex molecular mechanisms by which distinct flavanone compounds regulate adipocyte differentiation.

PE exerts well-known beneficial metabolic effects, such as protecting against several pathological conditions, including metabolic syndrome (MetS), neurodegenerative disorders, and cancer [10]. In the recent decades, the prevalence of obesity among children and adolescents has increased dramatically [11]. Popescu et al. (Contribution 3) conducted a prospective randomized study that involved 160 children with overweight or obesity in Romania, in which half of the participants underwent a 12-week kinesiotherapy program. This intervention significantly improved physical endurance parameters and decreased serum leptin and C-reactive protein levels, suggesting the alleviation of adiposity and inflammation, with a positive correlation observed between leptin levels and physical activity among participants. Boppre et al. (Contribution 4) further evaluated the correlations between body composition, muscle strength, and respiratory function parameters in 68 Portuguese firefighters. Anthropometric data collection, dual-energy X-ray absorptiometry, handgrip dynamometry, and spirometry were carried out for all participants. The most significant correlations were found between lean body mass and handgrip strength or forced vital capacity, suggesting that healthy adipose tissue phenotype and optimal body composition support physical endurance and respiratory functions, even in individuals with high energy demands.

Based on extensive research findings, pharmaceutical companies have started to develop drugs targeting key regulatory pathways involved in food intake, gastric emptying, and insulin release. Incretin analogs have demonstrated substantial efficacy not only in the treatment of diabetes mellitus but also in obesity management [12]. However, due to their potential adverse effects and limited availability, the development and application of alternative approaches are necessary [13]. Cryolipolysis, as one such approach, is a non-invasive method that induces adipocyte death through local cold shock, thereby reducing fat deposits in targeted body regions [14]. Lopes-Martins et al. (Contribution 5) conducted a study involving 30 female participants who underwent three abdominal cryolipolysis sessions; this intervention not only reduced body fat mass and body mass index but also improved serum total and low-density lipoprotein cholesterol levels, suggesting a potential reduction in the cardiovascular risk among patients with overweight or obesity through cryolipolysis.

## 3. Conclusions

Genetic predisposition, chronic overnutrition, and a sedentary lifestyle contribute to fat accumulation and adipose tissue dysfunction, supporting the multifactorial characteristics of polygenic obesity and its comorbidities. Excess energy intake coupled with insufficient energy expenditure leads to adipocyte hypertrophy, recruitment of MΦs and other immune cells, and chronic inflammation—particularly in visceral adipose tissue—ultimately resulting in insulin resistance and MetS. PE not only enhances energy catabolism to meet the high demand of muscle contractility but also promotes the release of myokines (termed as exerkines), thereby improving systemic glucose tolerance, promoting adipose tissue browning, and alleviating inflammation. The articles published in this Special Issue highlight emerging auxiliary therapeutic strategies, including the administration of herbal compounds, PE interventions, and cryolipolysis, targeting excess adiposity, inflammation, and metabolic dysregulation in obesity. Collectively, these observations emphasize the need for integrative and personalized approaches in the prevention and treatment of obesity and MetS.

## Data Availability

Not applicable.

## Abbreviations

The following abbreviations are used in this manuscript:

IL	Interleukin
MetS	Metabolic syndrome
MΦ	Macrophage
PE	Physical exercise
